# The Mucous Membrane of the Uterus after Parturition

**Published:** 1849-01

**Authors:** 


*The Mucous Membrane of the Uterus after Parturition.*—Various opinions prevail among accoucheurs relative to the state of the membrane lining the uterus after parturition ; some maintaining that a certain amount of the mucous membrane is left ; others, that shreds of the decidua remain ; and others, again, that the internal surface of the organ presents, after delivery, the denuded muscular fibres. Most writers on the subject agree, however, that the decidua is neither mediately or immediately cast off, but Burdach and Müller are not at all explicit on this question. Those accoucheurs who have principally derived their view from necroscopical appearances after puerperal fever, have described a sanious, putrilaginous, and pseudo-membranous layer, as forming the internal lining of the uterus after parturition. It would, however, appear, from the numerous and interesting investigations of Dr. Colin, mentioned in L'Union Médicale. 1. That in the normal state the internal surface of the uterus, after delivery, is not laid bare so as to expose the muscular fibres. 2. That even in cases of abortion there remains a vascular membranous layer. 3. That this latter is identical with the uterine layer of the decidua, and may therefore be looked upon as a mucous membrane of the uterus. 4. That this membrane is not entirely carried away by the lochia, whether the latter be sanguineous or purulent. 5. That a few particles previously detached may be cast off by the lochia, but that the part which is endowed with vitality remains. 6. That this latter part is the web upon which the reparatory process that is to re-constitute the uterine mucous membrane is conducted. 7. That the purulent and lactescent lochia, far from having upon that membrane the injurious effects which it is supposed to produce, are the simple results of the reparatory process. 8. That this membranous layer assumes all the characters of a mucous membrane towards the twentieth or thirtieth day after delivery. 9. That it is at first pulpy and thicker than in the normal state. And 10. That its elements gradually acquire more firmness, and that it resumes all its characters about the sixtieth or seventieth day after parturition.

The author had occasion, during the prevalence of puerperal fever at the Hôtel Dieu, in 1846, to examine twenty-two women who died of that disease. He generally observed the lining of the uterus very pulpy, and even slimy, or semi-fluid, towards the fundus of the organ; whilst towards the cervix it looked pseudo-membranous, vascular, and friable. When the alterations were more marked the coat lining the fundus, instead of being covered by a brown, pulpy substance, presented a greyish or greenish slough, and in some cases he even found the whole fundus quite denuded. The only symptom which Dr. Colin thinks indicative of the putrid softening of the uterine mucous layer is the fœtidity of the lochia. The total expulsion of the mucous membrane is not a constant phenomenon, when the patient recovers from puerperal fever; and it is an error, according to him, to suppose that the prominent disk, which gave insertion to the placenta, gets disorganized and expelled, at least, in the great majority of cases.

